# Supplementary material for: Pattern Pick and Place Method for Twisted Bi- and Multi-Layer Graphene
Source: Materials (Basel). 2019 Nov 13;12(22):3740. doi: 10.3390/ma12223740 (PMC6888300; doi:10.3390/ma12223740)
Supplement: Supplementary file 1 [file materials-12-03740-s001.pdf]

materials

Supplementary Material

Pattern Pick and Place Method for Twisted Bi- and Multi-Layer Graphene

Jae-Young Lim [1,§], Hyeon-Sik Jang [1,§], Hyun-Jae Yoo [1], Seung-il Kim [2] and Dongmok Whang [1,*]

[1] SKKU Advanced Institute of Nanotechnology (SAINT) and School of Advanced Materials Science and Engineering Sungkyunkwan University (SSKU), 2066, Seobu-Ro, Jangan-Gu, Suwon-Si, Gyeonggi-Do 16419, Korea; limjyyy@skku.edu (J.-Y.L.); dagu1821@skku.edu (H.-S.J); silverains2@gmail.com (H.-J.Y.)
[2] Department of Energy Systems Research and Department of Materials Science and Engineering Ajou University, 2016, World cup-Ro, Yeongtong-Gu, Suwon-Si, Gyeonggi-Do 16499, Korea; stmddlfs@gmail.com
* Correspondence: dwhang@skku.edu; Tel.: +82-31-290-7399
§ These authors contributed equally to this work.

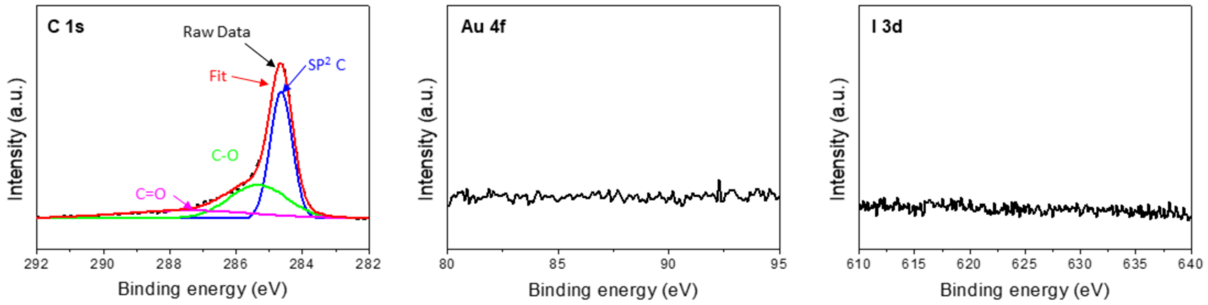

Figure S1. The XPSs results of tBLG at each C 1s, Au 4f, and I 3d.

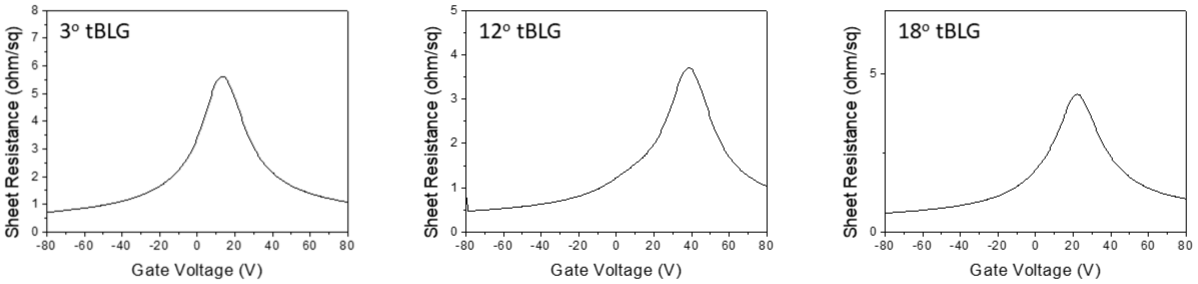

Figure S2. Back gate dependent sheet resistance graphs for 3°, 12°, and 18° tBLG devices.
